# Sociodemographic factors and health conditions associated with the resilience of people with chronic diseases: a cross sectional study**^1^**

**DOI:** 10.1590/1518-8345.1205.2786

**Published:** 2016-09-01

**Authors:** Julia Estela Willrich Böell, Denise Maria Guerreiro Vieira da Silva, Kathleen Mary Hegadoren

**Affiliations:** 2 Doctoral Student, Departamento de Enfermagem, Universidade Federal de Santa Catarina, Florianópolis, SC, Brazil. Substitute Professor, Departamento de Enfermagem, Universidade Federal de Santa Catarina, Florianópolis, SC, Brazil.; 3 PhD, Full Professor, Departamento de Enfermagem, Universidade Federal de Santa Catarina, Florianópolis, SC, Brazil.; 4 PhD, Full Professor, Faculty of Nursing, University of Alberta, Edmonton, Alberta, Canada.

**Keywords:** Resilience, Psychological, Chronic Disease, Nursing, Diabetes Mellitus, Renal Insufficiency, Chronic

## Abstract

**Objective:**

to investigate the association between resilience and sociodemographic variables and the health of people with chronic kidney disease and / or type 2 diabetes mellitus.

**Method:**

a cross-sectional observational study performed with 603 people with chronic kidney disease and / or type 2 diabetes mellitus. A tool to collect socio-demographic and health data and the Resilience Scale developed by Connor and Davidson were applied. A descriptive and multivariate analysis was performed.

**Results:**

the study participants had on average 61 years old (SD= 13.2), with a stable union (52.24%), religion (96.7%), retired (49.09%), with primary education (65%) and income up to three minimum wages. Participants with kidney disease showed less resilience than people with diabetes.

**Conclusion:**

the type of chronic illness, disease duration, body mass index and religious beliefs influenced the resilience of the study participants.

## Introduction

The changes that chronic disease brings are not always addressed properly, which may cause difficulties in the care and control of this disease, causing stress and bringing suffering to those persons and their families. However, it is realized that some of these people manage to overcome these difficulties keeping adherence to treatment and coping with their illness as something to be overcome, even if they often have many other problems in the different areas of their life. This may be related to the concept of resilience.

Resilience is still an understudied construct in the scientific literature in the field of nursing. However, in health care, it is being explored with studies, especially with traumatic situations and, more recently, studies of people with chronic health conditions^1^ ^-^ ^4^.

There are numerous definitions and approaches about what is resilience, guided by the complexity and the presence of factors and variables in studies of human phenomena. There is convergence about its connection to people who can overcome adversity or risk conditions, permeated by the interaction of biological and psychosocial conditions that result in positive adaptation allowing the development of the internal capacities of the person, as well as being considered to be a dynamic process^5^. When it comes to illness, resilience appears as a possibility of change, being understood as a person's ability to cope with illness, accepting the limitations imposed by the condition, with due adherence to treatment, seeking to adapt to the situation and live positively^6^. Resilience can enable some control over the negative impact of the physical, social and economic consequences, and the emotional consequences perceived in the disease ^7^. 

In this sense, the use of the concept of resilience can also be seen as a possibility to promote the acceptance of the indicated modifications and easier adaptation to the new health habits. Tackling chronic disease and adapting to the new lifestyle takes effort, dedication and to overcome the situation. Thus, resilience has proven to be a concept that can contribute to the control of chronic disease^1^ ^,^ ^3^ ^,^ ^8^ ^-^ ^9^. However, few studies have addressed the association of resilience, sociodemographic aspects and health condition of people with chronic diseases^7^ ^-^ ^9^. To show these variables in association with resilience can contribute to the development of a theoretical model that shows how resilience is expressed in chronic disease in order to collaborate in health care, indicating elements to promote better coping with the situation.

In this sense, we sought to know how resilience is expressed in these people and what aspects could influence it. Thus, it was defined as objective of the study: To assess the association between resilience and sociodemographic and health conditions of people with chronic kidney disease and / or type 2 diabetes mellitus.

## Method

A cross-sectional observational study conducted by the Center for Studies and Assistance in Nursing and Health to People in Chronic Condition (NUCRON - in Portuguese) with two distinct groups of people with chronic conditions, coming from two surveys that have in common the way of assessment of resilience, pertaining to the same macroproject research. A survey was conducted with men and women with chronic kidney disease (CKD) and the other survey with women with type 2 diabetes mellitus (DM2). This study used the database of the studies mentioned, in order to deepen the knowledge and identification of resilience in people with different chronic diseases.

The study involved people with CKD, men and women from four nephrology services in Greater Florianópolis / SC. We included all people registered in the services that met the study inclusion criteria, totaling 191 participants. The specific inclusion criteria were: minimum age of 18 years; to be in hemodialysis; to be capable to understand and communicate verbally, evaluated subjectively by the researchers. Data collection occurred in the months of May to October 2012.

The other study, which also composed the current study database, was conducted with women with DM2, registered in the Information System of Primary Health Care of Florianópolis Municipality, selected from a population of 1,820 records. To calculate the sample a 95% confidence interval was established, with a sampling error margin of 5%, the minimum amount being equivalent to 317. Given that the data provided by the Municipal Health could not be exact because the municipality did not present at the time of data collection 100% coverage in primary care, we sought to guarantee the data by expanding the sample in approximately 20%, totaling 412 participants. A stratification of the number of participants by each basic health unit and women with DM2 registered in these units was carried out, they were randomly selected by the online computerized tool SEstatNet^(r)^. The inclusion criteria were: minimum age of 18 years; to be capable to understand and communicate verbally; and to have received the diagnosis of DM2 for more than a year. People who have not been located after the third attempt by telephone or personal contact were excluded, being replaced by the next person in the list. The collection began in April 2010 and it was completed in July 2012. Therefore, the study sample consisted of 603 people.

Two PhD students and 12 nursing students, previously trained to carry out the data collection conducted the interviews, using standardized forms and procedures. Questionnaires containing sociodemographic and health information were used, and to assess the resilience we used the Resilience Scale developed by Connor and Davidson (CD-RISC)^10^, composed of 25 items in a *Likert* scale ranging from zero (not at all true) to four (almost always true), with scores ranging from zero to 100, with higher values indicating high resilience. The scale was evaluated regarding the internal consistency, test / retest reliability, convergent and discriminant validity and structural factor, presenting satisfactory psychometric properties, allowing distinction between people with higher and lower resilience^10^. This scale assesses five factors: personal competence; confidence in one's own instincts and tolerance to adversity; positive acceptance of change; control; and spirituality. The authors authorized the use of the scale, sending the version validated for the Brazilian population, designated as: Connor-Davidson Resilience Scale for Brazil (RISC-Br).

From the authorization of the authors of the research, the database of the study was organized in Excel^(r)^, Microsoft^(r)^. The independent variables analyzed and that composed the database were: age, religion, marital status, education, economic activity, monthly income, medical diagnosis of chronic disease, duration of disease, complications of the underlying disease, presence of hypertension systemic blood pressure (hypertension), presence of other illnesses and body mass index (BMI). For the latter variable, the height was evaluated with the use of a measuring tape with a semi soft graduation in centimeters (cm) and weight using a portable digital scale with pressure sensor with a maximum capacity of 150 kg, divided every 100 grams. 

The descriptive analyzes were performed through SEstatNet^(r)^, and the regressive multivariate analysis was performed through the Stata SE program 9.0(r), considering the mean of the resilience variable with the respective confidence intervals (CI) of 95% according to the independent variables. Since the dependent variable - Resilience - was of the discrete type, we used the count model with the Poisson regression for the gross and also for the adjusted analysis, so as to estimate how the changes in the independent variables affected the conditional mean and the probability count. The p value was estimated by the test F. In the adjusted analysis, the variables with p <0.20 in the gross analysis were included in the model, and the variables that reached p <0.05 and/or that fit the analysis, remained in the model.

All ethics precepts specified by the Resolution No. 196/96 of the National Health Council were respected, through the fulfillment of the requirements of the Free Informed Consent form. The study was submitted to the Ethics Committee of the Federal University of Santa Catarina, receiving authorization under protocol number 151/09 and FR 259792, related to the study with women with DM2, and the protocol 2118 and FR: 434113, concerning the study of people with CKD.

## Results

The study participants had on average 61 years old (SD = 13.2). Regarding gender 493 (81.8%) women and 110 (18.2%) men participated. Table 1 presents the sociodemographic characteristics, and Table 2 the health conditions for the total sample and by disease group. We emphasize that the variable "individual monthly income of the person" was answered by 368 people, considering the value of the minimum wage in the year of data collection; and the BMI variable was obtained from 577 people, since 26 did not accept and / or had not physical condition to perform this measurement. 

**Table 1:** Sociodemographic characterization of the study sample. Florianópolis, SC, Brazil, 2010-2012

Variable		People with DM2^*^ n^†^=412 n(%)	People with CKD^‡^ n^†^=191 n(%)	Total Sample n^†^=603 n(%)
Age group				
	Adult	129(32,0)	120(63,0)	249(41,3)
	Elderly	283(68,0)	71(37,0)	354(58,7)
Religion				
	No religion	14(3,3)	6(3,2)	20(3,3)
	With religion	398(96,7)	185(96,8)	583(96,7)
Marital Status				
	Without a partner	203(50,3)	85(44,5)	288(47,8)
	Stable Union	209(50,7)	106(55,5)	315(52,2)
Education				
	Never Studied	29(7,0)	12(6,0)	41(6,8)
	1st to 5th grade	284(69,0)	107(56,0)	312(51,7)
	6th to 8th grade	77(19,0)	39(21,0)	129(21,4)
	High School	3(1,0)	14(7,0)	36(6,0)
	Undergraduate	19(4,0)	19(10,0)	85(14,1)
Economically active				
	Do not Work	97(23,5)	27(14,0)	124(20,6)
	Work	100(24,3)	24(12,5)	124(20,6)
	Retired	215(52,2)	140(73,5)	355(58,8)
Monthly Income				
	Up to 1 MW^§^	125(42,0)	22(31,0)	22(6,0)
	>1 MW up to 3 MW	121(41,0)	34(47,0)	278(75,5)
	>3 MW	50(17,0)	16(22,0)	68(18,5)

*Diabetes Mellitus type 2; †total number; ‡Chronic Kidney Disease; §Minimum Wage, Brazil: R$510.00 (2010), R$545.00 (2011) and R$622.00 (2012)

**Table 2:** Health Determinants of the study sample. Florianópolis, SC, Brazil, 2010-2012

Variable		People with DM2^*^ (n^†^=412)	People with CKD^‡^ (n^†^=191)	Total Sample (n^†^=603)
Disease time in years				
	Mean (SD)^§^	10,7 (8,4)	8,3 (6,7)	10,0 (8,0)
Complications				
	Yes	33,0%	57,0%	41,0%
	No	67,0%	43,0%	59,0%
Other Diseases				
	Yes	96,0%	57,0%	79,0%
	No	4,0%	43,0%	21,0%
Hypertension^||^				
	Yes	78,0%	77,0%	77,0%
	No	22,0%	23,0%	23,0%
BMI				
	Mean (SD)^§^	30(5,5)	23,3(5,8)	28,1(6,3)

*Diabetes Mellitus type 2; †total number; ‡Chronic Kidney Disease; §Standard deviation; ||Hypertension; ¶Body mass index

The resilience of people with chronic diseases had a mean score of 76.2 (SD = 14.7), with an occurrence of significant variation in scores, with a minimum of 25 and maximum of 100. People with DM2 had a mean score of resilience equal to 79.8 (SD = 12.9) higher than people with CKD, with a mean resilience of 67.5 (SD = 15.4). 

Through the results of the adjusted analysis of the average ratio it was evident that the variables that influence the resilience in this study were: disease duration, type of chronic disease, religion and BMI. Regarding the *duration of disease*, people with six to 10 years of disease and over 16 years of disease had the lowest resilience scores. Having a *religious belief* impacts positively on resilience, meaning that people who claimed to have religion had a better mean score of resilience. Regarding *BMI*, people with obesity grade I showed better resilience than others (Table 3).

**Table 3:** Multivariate analysis of sociodemographic characteristics and resilience of people with chronic diseases. Florianópolis, SC, Brazil, 2010-2012

Variables		Mean Resilience (IC* 95%)	Ratio of Gross Income† (IC95%)	Value p‡	Average Ratio Adjusted§ (IC95%)
Gender				<0,001	
	Feminine	77,9 (76,7-79,1)	1,13 (1,10-1,16)		1,02 (0,96-1,08)
	Masculine	68,9 (65,7-72,2)	1		1
Age Group				0,008	
	Adult	75,1 (73,3-77,0)	1		1
	Elderly	77,0 (75,5-78,6)	1,03 (1,01-1,04)		1,02 (0,98-1,05)
Chronic Disease				<0,001	
	DM2^||^	79,8 (78,5-81,0)	1		1
	CKD^¶^	67,5 (64,8-70,2)	0,85 (0,83-0,87)		0,85 (0,80-0,90)
	DM2^||^ and CKD^¶^	72,5 (68,8-76,2)	0,91 (0,88-0,94)		0,97 (0,91-1,03)
Religion				0,001	
	No religion	70,3 (61,2-79,4)	1		1
	With religion	76,5 (75,3-77,6)	1,09 (1,03-1,15)		1,13 (1,06-1,20)
Marital Status				0,182	
	Without a partner	76,7 (75,1-78,5)	1		1
	Married/stable union	75,8 (74,2-77,5)	0,98 (0,97-1,01)		0,98 (0,95-1,00)
Education				0,005	
	Never studied	75,4 (71,3-79,6)	1		1
	1st to 5th grade	76,8 (75,2-78,4)	1,02(0,98-1,06)		0,99 (0,94-1,03)
	6th to 8th grade	76,4 (73,7-79,1)	1,01 (0,97-1,05)		1,02 (0,97-1,08)
	High School	78,9 (75,1-82,8)	1,04 (0,99-1,10)		1,04 (0,97-1,12)
	Undergraduate	73,3 (69,9-76,9)	0,97 (0,93-1,01)		0,96 (0,90-1,02)
Economically Active				<0,001	
	Doesn't work	77,3 (74,8-79,7)	1		1
	Works	80,0 (77,7-82,4)	1,04 (1,01-1,06)		0,98 (0,92-1,04)
	Retired	74,6 (73,0-76,2)	0,96 (0,94-0,99)		0,96 (0,91-1,01)
Income				0,145	
	To 1 MW^**^	78,6 (72,7-84,5)	1		1
	>1 MW to 3 MW	78,5 (76,9-80,1)	0,99 (0,95-1,05)		0,99 (0,94-1,06)
	>3 MW	76,2 (72,2-80,2)	0,97 (0,92-1,02)		0,96 (0,90-1,02)
Duration of Disease				0,014	
	Until 5 years	77,0 (75,1-79,0)	1		1
	6 - 10 years	75,2 (72,9-77,4)	0,98 (0,95-0,99)		0,95 (0,92-0,98)
	11 - 15 years	74,5 (71,5-77,5)	0,96 (0,93-0,99)		0,97 (0,94-1,01)
	>16 years	77,6 (75,0-80,2)	1,01 (0,98-1,03)		0,95 (0,91-0,98)
Complications				<0,001	
	No	77,5 (76,0-79,0)	1		1
	Yes	74,5 (72,7-76,3)	0,96 (0,94-0,98)		1,00 (0,97-1,03)
Hypertension^††^				0,254	
	No	75,5 (73,0-78,0)	1		
	Sim	76,5 (75,1-77,8)	1,01 (0,99-1,03)		
Other Diseases				<0,001	
	No	68,7 (65,8-71,5)	1		1
	Yes	78,2 (77,0-79,5)	(1,11-1,16)		1,03 (0,98-1,08)
BMI^‡‡^				<0,001	
	Ideal Weight	71,3 (69,2-73,4)	1		1
	Overweight	76,8 (74,6-79,1)	1,08 (1,05-1,10)		1,03 (0,99-1,08)
	Obesity grade I	81,6 (79,6-83,7)	1,14 (1,11-1,17)		1,06 (1,02-1,09)
	Obesity grade II	79,7 (76,1-83,2)	1,12 (1,08-1,16)		1,03 (0,98-1,09)
	Morbid Obesity	80,2 (74,6-85,8)	1,12 (1,07-1,18)		1,03 (0,98-1,09)

* Confidence Interval; † Count model with *Poisson* regression*.;* ‡ Estimated pelo teste F.; § Count model with *Poisson* regression ; ||Diabetes Mellitus type 2

¶Chronic Kidney Disease; **Minimum Wage, Brazil: R$510.00 (2010), R$545.00 (2011) and R$622.00 (2012); ††Hypertension; ‡‡Body Mass Index

## Discussion

From the adjusted multivariate analysis it was found that, among the socio-demographic factors, only religion influenced the resilience. Few authors relate resilience with religiosity, faith or spirituality. Religious belief is identified as a source of support to face difficult situations^7^. The other sociodemographic variables presented in our study - age, sex, marital status, labor activity, education and income - have not been identified as factors that influence resilience. With regard to age, in two other studies, one of them also developed with people with DM^8^ ^)^ and another with nurses^11^, a relationship between this variable and resilience was also not identified^8^ ^,^ ^11^. 

It is realized that living with chronic disease requires adjustment both for those living with diabetes^12^ and for those living with CKD^13^. In this study, the influence of resilience scores were related to health factors, such as type of chronic illness, disease duration and BMI.

In regards to the type of disease, diabetes is related to better resilience, which can be explained because it is a disease, often silent, which does not require sharp changes in people's lives. It does not present itself with physical and emotional manifestations, such as those caused by kidney disease, particularly for people who are undergoing hemodialysis. This specific way in how disease affects the lives of people with CKD may be the reason for the lower scores of resilience in those people^14^ ^-^ ^15^. Furthermore, depression, frequent in individuals with CKD, is a variable that can affect resilience^16^ ^-^ ^17^. This finding indicates the need for further research exploring resilience with other variables, such as depression.

Disease duration negatively influenced resilience in two periods: those participants presenting six to 10 years of disease, as well as those over 16 years, ie, the model of this study revealed through the adjusted analysis, those people in these disease duration ranges showed lower resilience scores. Studies of resilience mostly do not focus on the relationship with the duration of the disease. However, the increase of these diseases and complications arise from non-adherence over the years^12^ ^-^ ^14^. This means that the longer the time living with the disease, the larger the impact on people's lives, leading to lower resilience scores, as was obtained in our study.

As for the BMI, people with CKD maintained their weight within the normal parameter, which is associated with weight loss, one of the characteristic manifestations of this disease in its terminal stages^18^. One of the factors contributing to the weight loss is the necessity of the diet to which people on hemodialysis are submitted^18^.

Conversely, people with DM2 have low adherence to dietary restrictions, as well as this disease having obesity as one of its causes^19^. That means that overweight and obesity are common among people with DM. A previous study showed the prevalence of low adherence to the diet and physical inactivity in people with diabetes^20^. Also, it was found in another study that 55.78% of the women studied did not diet and 61.22% did not exercise, showing that specific care recommendations are not always followed^12^.

Regarding the association between resilience, marital status and sex, the findings obtained corroborate other studies, which also showed no significant correlation between these variables^10^ ^,^ ^21^ ^-^ ^22^. We believe that these variables should be explored in future studies. 

Regarding the variable education, we observed that resilience scores do not seem to be related to education, as even those who never studied have similar scores, meaning that being resilient is independent of the level of education as the other studies show^11^ ^,^ ^23^.

People who worked scored higher in resilience; however, the adjusted analysis demonstrated no influence of this variable in resilience, as well as income. Another study showed a different situation, with income significantly related to resilience^8^. There are few studies that relate resilience to economic status. However, one author states that having a low socioeconomic status does not prevent the development of resilience^1^.

Studies of people with DM and that used the CD-RISC to analyze the resilience found average scores ranging from 74.9 (SD = 14.8) and 83.1 (SD = 8.5)^22^ ^-^ ^23^. In another study, also with people with diabetes, but using a different resilience scale (RS from Wagnild and Young), findings showed that more than half of the participants (66.4%) were classified as highly resilient, through the mean and standard deviation found in the study^8^. These findings also concur with the results of the present study, stressing that people with diabetes have shown high scores of resilience, i.e. very close to those of healthy people^10^.

Despite the fact that the research on resilience of people with CKD are scarce, what we have observed is that the findings refer to smaller resilience scores for this population, as presented in the study that applied the Wagnild and Young's Resilience Scale^24^. Studies done with the CD-RISC that found lower scores for resilience, approaching those found in people with CKD, were those in people with depression, psychiatric anxiety disorders and schizophrenia and those that suffered some kind of trauma. The scores in those studies were between 46.1 (SD = 18.7) and 68.0 (SD = 15.3) points^10^ ^,^ ^25^. 

The differences between the two groups may be considered a limitation of the study, especially due to the fact that one study was conducted only with women with DM2. However, this limitation can be relativized by the fact that comparing the groups in regards to sociodemographic data was not a goal of the study, but it was rather to verify the association between resilience and sociodemographic and health conditions of people with chronic kidney disease and/or diabetes mellitus type 2. In addition, the adjusted analysis identified no difference between men and women's resilience scores. 

## Conclusions

The study revealed that resilience is associated with the type of chronic disease, i.e., people with CKD on hemodialysis have lower resilience scores than people with DM. Along with the type of disease, the duration of disease, BMI and having a religion were also factors that influence the resilience of people with these chronic diseases.

Given the undeniable need to find options to help to better control chronic diseases and to achieve a more harmonious relationship with them, the concept of resilience emerges as a possibility still needing further studies, exploring the association with other variables, as well as the evaluation of strategies for promoting resilience.

## References

[1] Newton-John TR, Mason C, Hunter M (2014). The role of resilience in adjustment and coping with chronic pain. Rehabil Psychol.

[2] Tian J, Hong J (2014). Assessment of the relationship between resilience and quality of life in patients with digestive cancer. Wld J Gastroenterol: WJG.

[3] Slomka L. (2011). Associação entre o nível de resiliência e o estado clínico de pacientes renais crônicos em hemodiálise. Barbarói.

[4] Yi-Frazier JP, Smith RE, Vitaliano PP, Yi JC, Mai S, Hillman M (2010). A Person-Focused Analysis of Resilience Resources and Coping in Diabetes Patients. Stress Health.

[5] Rutter M (2012). Resilience as a dynamic concept. Develop Psychopathol.

[6] Bianchini DCS, Dell'aglio DD (2006). Processos de resiliência no contexto de hospitalização: um estudo de caso. Paidéia.

[7] Vinaccia SJ, Quiceno JM, Remor E (2012). Resiliencia, percepción de enfermedad, creencias y afrontamiento espiritual-religioso en relación con la calidad de vida relacionada con la salud en enfermos crónicos colombianos. Anales Psicología.

[8] Denisco S (2011). Exploring the relationship between resilience and diabetes outcomes in African Americans. J Am Acad Nurse Pract.

[9] Nawaz A, Malik JA, Batool A (2014). Relationship between resilience and quality of life in diabetics. J Coll Physicians Surg Pak.

[10] Connor KM, Davidson JRT (2003). Development of a new Resilience Scale: The Connor-Davidson Resilience Scale (Cd-Risc). Depress Anxiety.

[11] Gillespie BM, Chaboyer W, Wallis M (2009). The influence of personal characteristics on the resilience of operating room nurses A predictor study. Int J Nurs Stud.

[12] Lessmann JC, Silva DMGV, Nassar SM. (2012). Mulheres com Diabetes mellitus tipo 2: perfil sociodemográfico, biométrico e de saúde. Acta Paul Enferm.

[13] Coutinho NOS, Tavares MCHT. (2011). Atenção ao paciente renal crônico, em hemodiálise, sob a ótica do usuário. Cad Saúde Coletiva.

[14] Terra FS, Costa AMDD, Ribeiro CCS, Nogueira CS, Prado JP, Costa MD (2010). O portador de insuficiência renal crônica e sua dependência ao tratamento hemodialítico: compreensão fenomenológica. Rev Bras Clin Med.

[15] Cardoso LB, Sade PMC (2012). O enfermeiro frente ao processo de resiliência do paciente em tratamento hemodialítico. Rev Eletr Faculdade Evangélica do Paraná.

[16] Halen NV, Cukor D, Constantiner M, Kimmel PL (2012). Depression and mortality in end-stage renal disease. Curr Psychiatry Rep.

[17] Griffiths FE, Boardman FK, Chondros P, Dowrick CF, Densley K, Hegart K L (2015). The effect of strategies of personal resilience on depression recovery in an Australian cohort A mixed methods study. Health.

[18] Molnar MZ, Streja E, Kovesdy CP, Bunnapradist S, Sampaio MS, Jing J (2011). Associations of Body Mass Index and Weight Loss with Mortality in Transplant-Waitlisted Maintenance Hemodialysis Patients. Am J Transplant.

[19] American Diabetes Association (ADA) (2015). Standards of Medical Care in Diabetes. Diabetes Care.

[20] Boas LCGV, Foss MC, Foss-Freitas MC, Torres HDC, Monteiro LZ, Pace AE (2011). Adesão à dieta e ao exercício físico das pessoas com diabetes mellitus. Texto Contexto Enferm.

[21] Fortes TFR, Portuguez MW, Argimon IIL. (2009). A resiliência em idosos e sua relação com variáveis sociodemográficas e funções cognitivas. Estud Psicol. (Campinas).

[22] Tavares BC, Barreto FA, Lodetti ML, Silva DMGV, Lessmann JC. (2011). Resiliência de pessoas com Diabetes Mellitus. Texto Contexto - Enferm..

[23] Steinhardt MA, Mamerow MM, Brown SA, Jolly CA. (2009). A Resilience Intervention in African American Adults with Type 2 Diabetes: A Pilot Study of Efficacy. Diabetes Educ.

[24] Ma LC, Chang HJ, Liu YM, Hsieh HL, Lo L, Lin MY (2013). The relationship between health-promoting behaviors and resilience in patients with chronic kidney disease. Sci World J. [Internet] 2013. [Acesso 10 jan 2015];.

[25] Min JA, Lee NB, Lee CU, Lee C, Chae JH (2012). Low trait anxiety, high resilience, and their possible interaction as predictors for treatment response in patients with depression. J Affect Disord.

